# Establishing Co-Culture Blood–Brain Barrier Models for Different Neurodegeneration Conditions to Understand Its Effect on BBB Integrity

**DOI:** 10.3390/ijms24065283

**Published:** 2023-03-09

**Authors:** Jun Sung Park, Kyonghwan Choe, Amjad Khan, Myeung Hoon Jo, Hyun Young Park, Min Hwa Kang, Tae Ju Park, Myeong Ok Kim

**Affiliations:** 1 Division of Life Science and Applied Life Science (BK21 FOUR), College of Natural Sciences, Gyeongsang National University, Jinju 52828, Republic of Korea; 2 Department of Psychiatry and Neuropsychology, School for Mental Health and Neuroscience (MHeNs), Maastricht University, 6229 ER Maastricht, The Netherlands; 3 Department of Pediatrics, Maastricht University Medical Center (MUMC+), 6202 AZ Maastricht, The Netherlands; 4 Haemato-Oncology/Systems Medicine Group, Paul O’Gorman Leukaemia Research Centre, Institute of Cancer Sciences, MVLS, University of Glasgow, Glasgow G12 0ZD, UK; 5 Alz-Dementia Korea Co., Jinju 52828, Republic of Korea

**Keywords:** BBB, in vitro model, TEER, brain endothelial cells, astrocytes, tight junction proteins, neurodegenerative diseases

## Abstract

The blood–brain barrier (BBB) is a functional interface that provides selective permeability, protection from toxic substances, transport of nutrients, and clearance of brain metabolites. Additionally, BBB disruption has been shown to play a role in many neurodegenerative conditions and diseases. Therefore, the aim of this study was to establish a functional, convenient, and efficient in vitro co-cultured BBB model that can be used for several physiological conditions related to BBB disruption. Mouse brain-derived endothelial (bEnd.3) and astrocyte (C8-D1A) cells were co-cultured on transwell membranes to establish an intact and functional in vitro model. The co-cultured model and its effects on different neurological diseases and stress conditions, including Alzheimer’s disease (AD), neuroinflammation, and obesity, have been examined by transendothelial electrical resistance (TEER), fluorescein isothiocyanate (FITC) dextran, and tight junction protein analyses. Scanning electron microscope images showed evidence of astrocyte end-feet processes passing through the membrane of the transwell. Moreover, the co-cultured model showed effective barrier properties in the TEER, FITC, and solvent persistence and leakage tests when compared to the mono-cultured model. Additionally, the immunoblot results showed that the expression of tight junction proteins such as zonula occludens-1 (ZO-1), claudin-5, and occludin-1 was enhanced in the co-culture. Lastly, under disease conditions, the BBB structural and functional integrity was decreased. The present study demonstrated that the co-cultured in vitro model mimicked the BBB’s structural and functional integrity and, under disease conditions, the co-cultured model showed similar BBB damages. Therefore, the present in vitro BBB model can be used as a convenient and efficient experimental tool to investigate a wide range of BBB-related pathological and physiological studies.

## 1. Introduction

Homeostasis of the brain is maintained by a specialized structure called the blood–brain barrier (BBB) [1]. Along with the homeostasis of the brain, the BBB performs several essential and specialized functions, as acting as a cellular barrier and promoting simple diffusion in carrier-mediated transport, receptor-mediated transcytosis, active efflux through ATP-binding cassette (ABC) transporters, and the clearance of neurotoxic substances [2]. Recent evidence has suggested that BBB disruption is an early biomarker for cognitive decline and dementia-associated diseases [3].

The BBB is a complex three-dimensional structure comprised of brain endothelial cells, astrocytes, and pericytes [4]. Prolonged astrocyte structures called astrocytic end-feet cover endothelial cells to maintain the BBB [5]. The barrier nature of this complex is maintained by specialized brain endothelial cells, which form a compact junctional complex including tight junctions and adherens junctions [6]. Tight junction structures include transmembrane proteins (occludin, claudins and junctional adhesion molecules), actin filaments, and cytoplasmic scaffold proteins (zonula occluden (ZO)) [7]. Studies have shown that tight junction-related BBB dysfunction is associated with Alzheimer’s disease [8,9]. The expression of tight junction proteins during cell-to-cell contact leads to the formation of a compact barrier-like property. This results in enhanced transendothelial electrical resistance (TEER) and low transcellular and paracellular permeability, which restricts the lateral diffusion of membrane proteins, but allows transport through a specialized transport system [10,11,12].

Normal functioning of the BBB is essential for the proper and regulated functioning of the central nervous system (CNS). However, any disruption or dysregulation in the BBB can lead to severe neurological disorders. Several studies have shown that the disruption of the BBB is involved in the onset of Alzheimer’s disease (AD) [13,14,15]. Moreover, neuroinflammation has shown to damage the BBB and cause further development in AD [16]. Additionally, several peripheral metabolic disorders, such as hyperglycemia, diabetes, and cardiovascular disease, are known to be responsible for the disruption of the BBB. Peripheral metabolic disorders also challenge the integrity of the BBB [17]. A high-fat diet is a significant risk factor for the development of metabolic-related disorders such as hyperglycemia, diabetes, and cardiovascular disease and neuroinflammation [18,19,20]. Palmitic acid (PA) is the most common form of saturated fatty acid in nature, including the human body, and is rich in high-fat diets [21]. Additionally, these metabolic-related disorders and neuroinflammation are risk factors for developing dementia, including Alzheimer’s disease (AD) [22,23,24]. Furthermore, studies have also shown a direct association between PA and AD [25,26,27].

Due to the crucial role of the BBB in neurological disease and pharmacological research, a BBB model is an essential component. Currently, several BBB models have been developed, ranging from mono- and co-cultured models to sophisticated models, such as spheroid and chip models that use animal and human cell types [28,29,30,31,32]. Sophisticated models represent the BBB structure using the 3D organization of cells and mimic cerebral blood flow [31,32]. Despite these advantages, they are difficult to set up and, most importantly, expensive. Therefore, a co-cultured transwell system provides a simpler, cost-effective, and functional BBB model. Therefore, the present study describes a contact co-culture in vitro model that uses mouse brain endothelial and astrocyte cell lines in transwells to provide an efficient and functional platform for various neurological research scenarios.

## 2. Results

### 2.1. Endothelial and Astrocyte Co-Culture Model of the BBB Shows Efficient Inter-Cellular Communication by End-Feet Processes

**Figure 1:**
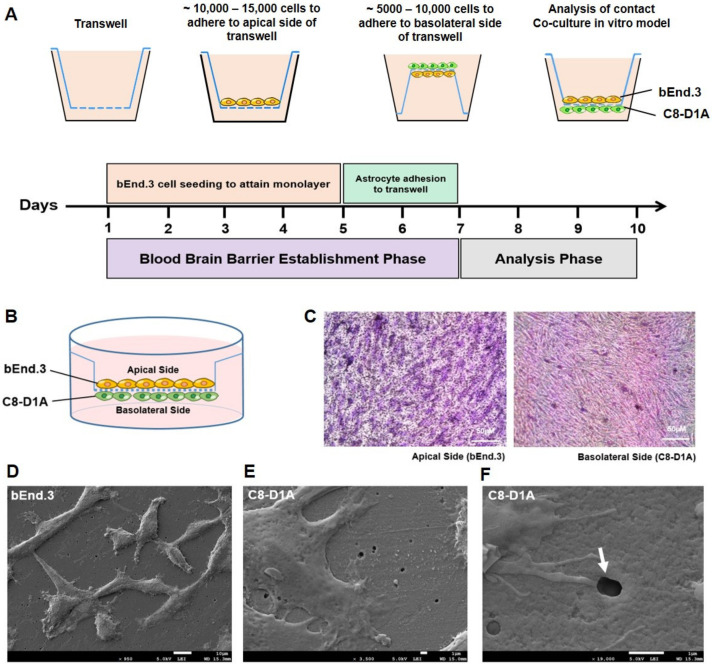
The establishment of a co-culture in vitro BBB model. (**A**) Schematic timeline representation of bEnd.3 and C8-D1A cell seeding on apical and basolateral sides of the transwell to attain a contact co-culture in vitro BBB model. (**B**) Graphical representation of intact contact co-culture in vitro BBB model. (**C**) Hematoxylin and eosin (H&E) stained transwell membrane shows adherent bEnd.3 and C8-D1A cells on the apical and basolateral side. (**D**–**F**) Scanning electron micrographs of transwell membrane at low confluency of cells show (**D**) bEnd.3 cells on the apical side (×950) and (**E**) C8-D1A cells on the basolateral side of the transwell membrane (×3500). (**F**) Magnified micrograph (×19,000) shows C8-D1A cell end-feet processes on the basolateral side passing through 0.4 µm pore (white arrow) to reach bEnd.3 cells on the apical side of the transwell membrane.

The brain endothelial cell line bEnd.3 and the astrocyte cell line C8-D1A were co-cultured on the transwells. The brain endothelial cells were cultured on the apical side, while C8-D1A cells were seeded on the basolateral side of the transwell during contact co-culture (Figure 1A–C). Cells that adhered to opposite sides of the membrane showed intercellular communication via protruded end-feet processes through the micropores of the membrane. Scanning electron microscope images showed evidence of the astrocyte end-feet processes passing through the membrane of the transwell (Figure 1D,F). This property mimics the actual BBB, where the astrocyte end-feet comes in contact with the endothelial cells (Figure 1F).

### 2.2. Co-Cultured BBB Model Showed Effective Barrier Function and Property

**Figure 2:**
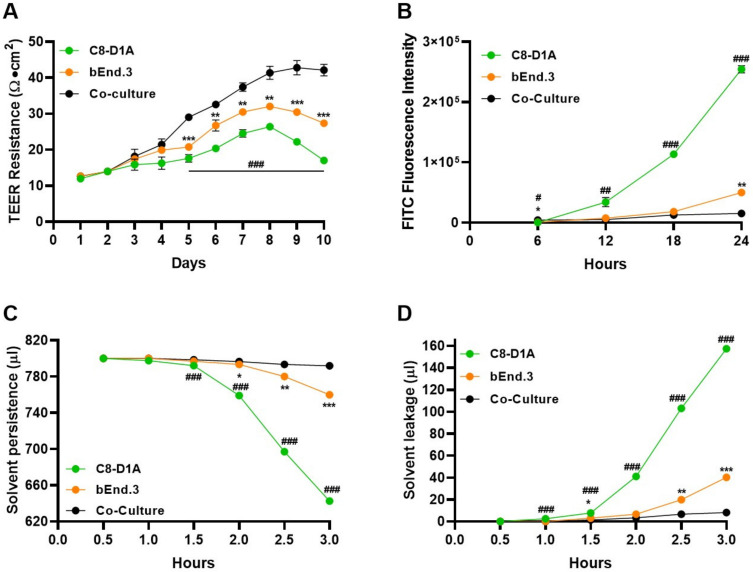
The co-culture BBB model showed efficient barrier properties. Assays were used to analyze the barrier property of bEnd.3 and C8-D1A cells seeded individually or grown in co-culture conditions. (**A**) Transendothelial electrical resistance (TEER) analysis using chopstick electrodes to record resistance across the transwell membrane grown with bEnd.3, C8-D1A cells or co-cultured. (**B**) Fluorescein isothiocyanate (FITC) dextran permeability assay was used to analyze the barrier capacity of the in vitro BBB model. (**C**) Solvent persistence and (**D**) solvent leakage tests were used to measure how long bEnd.3, C8-D1A, and co-cultured cells can hold the media on the apical side of the transwell. All data had a sample size of three (*n* = 3) and three experimental repeats. Data are presented as mean ± SEM. The mono-culture models were compared to the co-culture models (* bEnd.3, # C8-D1A). *p* < 0.05 was considered to be statistically significant. *^;#^ *p* < 0.05, **^;##^ *p* ≤ 0.01 and ***^;###^ *p* ≤ 0.001.

After establishing a co-cultured BBB model, we examined its barrier function and property (Figure 2). First, TEER values for both mono- and co-cultured C8-D1A and bEnd.3 cells were analyzed (Figure 2A and Figure S1A). The TEER values depict a functionally compact layer of cells that resist permeability. The co-cultured (C8-D1A and bEnd.3) transwell showed a greater TEER value than the individual cell cultured (C8-D1A or bEnd.3) transwells, which showed peak values from 5 to 8 days post-seeding. Additionally, the TEER value increased with time due to the confluency of cells, indicating that the co-culture transwell showed maximum resistance compared to the cells cultured individually. Hence, the present co-culture in vitro model replicates the quality of an intact BBB. Furthermore, it has been reported that the BBB is impermeable to molecules larger than 400 Da [33]. To check the integrity of the co-culture BBB model, an FITC-dextran 70 permeability assay was performed. The FITC fluorescence values showed that the co-cultured model resisted the penetration of FITC dextran better than the individual cell cultured models (Figure 2B and Figure S1B). Next, the structural aspect of the present contact co-culture model was analyzed by solvent persistence and solvent leakage tests. The results showed that the co-cultured transwells had the highest solvent persistence and lowest leakage values compared to the individual cell cultured models (Figure 2C,D and Figure S1C,D).

### 2.3. The Co-Cultured Model Showed Enhanced Cell–Cell Adhesions

**Figure 3:**
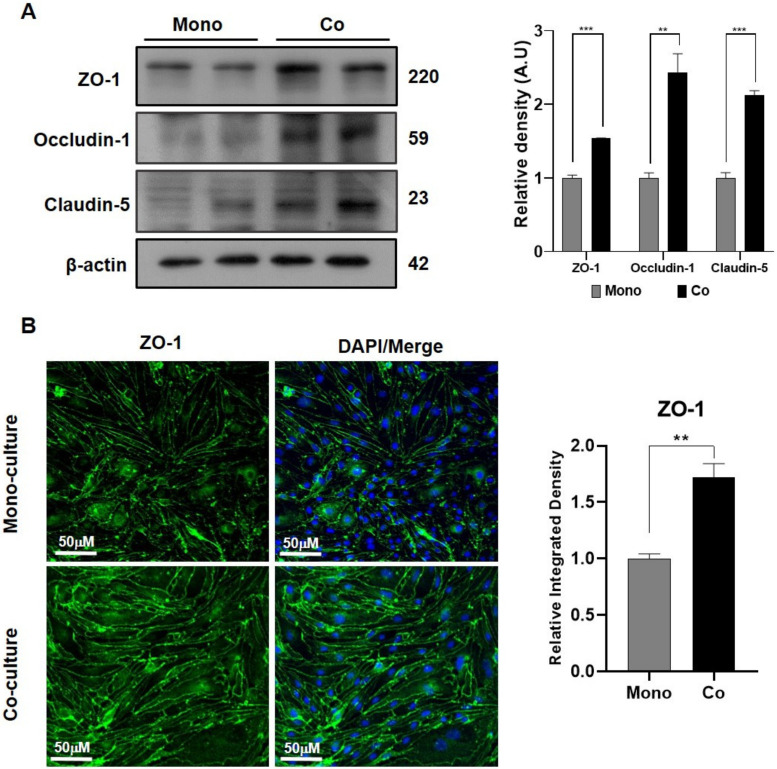
Brain endothelial cells in co-culture showed enhanced tight junction protein expression. (**A**) Immunoblot analysis was used to compare the expression levels of the tight junction proteins ZO-1, occludin-1, and claudin-5 protein in bEnd.3 cells between the monolayer and in the co-culture along with C8-D1A cells. (**B**) Immunofluorescence staining was used to analyze ZO-1 expression in bEnd.3 cells cultured in mono- and co-culture states. All data had a sample size of three (*n* = 3) and three experimental repeats. Data are presented as mean ± SEM. *p* < 0.05 was considered to be statistically significant. ** *p* ≤ 0.01 and *** *p* ≤ 0.001.

The expression of tight junction proteins such as ZO-1, occludin-1, and caludin-5 were analyzed in both mono- (without C8-D1A) and co- (with C8-D1A) cultured models to assess the cell–cell adhesions. The Western blot analysis showed that all the aforementioned tight junction protein expressions were 1.5–2-fold higher in the co-cultured model than in the mono-cultured model (Figure 3A). Additionally, the fluorescence immunohistochemistry analysis validated this finding by showing enhanced ZO-1 expression in the co-cultured model compared to the mono-cultured models (Figure 3B).

### 2.4. Exposure to Palmitic Acid Mimics Obesity and Distresses the BBB Tight Junction

**Figure 4:**
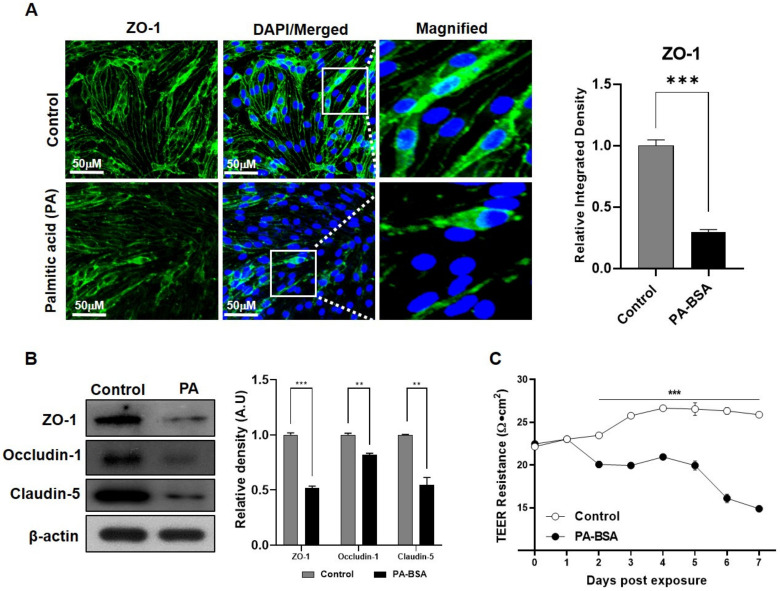
PA-BSA affects the expression of tight junction proteins. (**A**) Confocal microscope images show the co-expression of ZO-1 in bEnd.3 cells in the control and the bovine serum albumin-conjugated palmitic acid (PA-BSA)-treated group. (**B**) Immunoblot analysis was used to compare the expression levels of tight junction proteins ZO-1, occludin-1, and claudin-5 expressed in bEnd.3 cells from the control and PA-BSA-treated group. (**C**) TEER resistance values were measured across the co-cultured transwell membrane of the control group and PA-BSA-treated group. All data had a sample size of three (*n* = 3) and three experimental repeats. Data are presented as mean ± SEM. *p* < 0.05 was considered to be statistically significant. ** *p* ≤ 0.01 and *** *p* ≤ 0.001.

The in vitro obesity model was established by exposing PA-BSA in the co-cultured model for 24 h. The immunofluorescence results showed that there was a significant decline in ZO-1 in the PA-BSA-exposed co-culture model (Figure 4A). In the Western blot analysis, tight junction proteins, including ZO-1, occludin-1, and claudin-5, also showed a significant decline in the expression levels among PA-BSA-treated cells (Figure 4B). Moreover, the deteriorating effect of PA-BSA on the BBB’s integrity was analyzed by measuring the electrical resistance across the BBB. The TEER analysis further validated that PA-BSA exposure significantly deteriorated the electrical resistance of the BBB, thus indicating a weakened BBB structure (Figure 4C).

### 2.5. Neuroinflammation Affects the Decline in BBB Permeability

**Figure 5:**
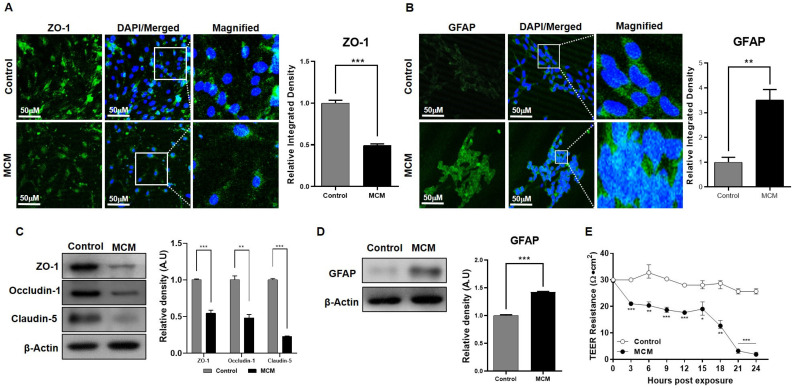
The effect of the pro-inflammatory response on the BBB. (**A**) Immunofluorescence staining was used to estimate the expression level of ZO-1 in bEnd.3 cells between the control and bEnd.3 cells exposed to activated microglial conditioned media (MCM). (**B**) Confocal microscopic images of C8-D1A cells compare glial fibrillary acidic protein (GFAP) expression in the control group as well as in the MCM-treated group. (**C**) Immunoblots show the protein expression of ZO-1, occuludin-1, and claudin-5 in bEnd.3 cells harvested from the control and MCM-exposed cells (**D**). Western blot comparison of GFAP protein expression in C8-D1A cells harvested from the control and MCM-exposed cells. (**E**) Comparison of TEER resistance values compared across the transwell membrane of the control and MCM-treated group. All data had a sample size of three (*n* = 3) and three experimental repeats. Data are presented as mean ± SEM. *p* < 0.05 was considered to be statistically significant. * *p* < 0.05, ** *p* ≤ 0.01 and *** *p* ≤ 0.001.

Neuroinflammation includes the microglial activation responsible for the secretion of interleukins and cytokines. To mimic this condition, our co-cultured model was exposed to microglial conditioned media (MCM), which is enriched with pro-inflammatory interleukins and cytokines. After 24 h of exposure, the immunofluorescence analysis showed that ZO-1 expression declined and glial fibrillary acidic protein (GFAP), a marker of astroglial injury, expression significantly increased in the MCM-treated group, as compared to the control group (Figure 5A,B). We validated this finding through western blot analysis and showed that the expression of tight junction proteins after MCM exposure significantly declined, while GFAP expression increased compared to the non-exposed control group (Figure 5C,D). Lastly, BBB integrity was analyzed using TEER analysis and showed a rapid decline in barrier capability after exposure to MCM (Figure 5E).

### 2.6. Amyloid Beta Pathology Leads to BBB Disruption

**Figure 6:**
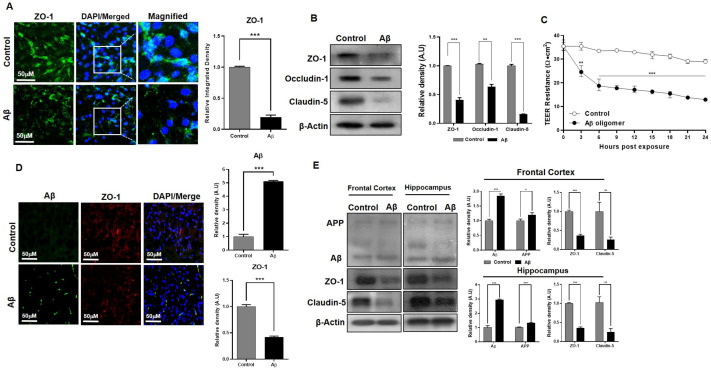
Amyloid beta toxicity in brain endothelial cells and astrocytes. (**A**) Confocal microscope images show the immunofluorescence of ZO-1 protein expression in control cells and amyloid beta (Aβ) (1–42) peptide-treated bEnd.3 cells. (**B**) Immunoblot analysis of the expression of tight junction proteins ZO-1, occludin-1, and claudin-5 in bEnd.3 cells harvested from control and amyloid beta (1–42) peptide-treated cells. (**C**) TEER analysis of resistance measured across transwell membrane with co-culture treated with Aβ peptides and triple co-culture with HT22 cells transfected with pCAX APPswe/ind plasmids. TEER values were compared with untreated control cells seeded on transwells. (**D**) Immunofluorescence analysis of Aβ and ZO-1 expression in the cerebral cortex region of the control and Aβ (1–42)-injected mice. (**E**) Western blot images show the protein expression of Aβ, APP, ZO-1, and claudin-5 in the frontal cortex and hippocampus of the control and Aβ-injected mice. All data had a sample size of three (*n* = 3) and three experimental repeats. Data are presented as mean ± SEM. *p* < 0.05 was considered to be statistically significant. * *p* < 0.05, ** *p* ≤ 0.01 and *** *p* ≤ 0.001.

An in vitro model for AD-like pathology with three co-cultured cell lines was created with bEnd.3 cells that were seeded on the apical side of the transwell, C8-D1A cells that were seeded on the basolateral side of the same transwell, and HT22 cells that were seeded in the wells of the plate. HT22 cells were transfected with the amyloid precursor protein overexpression plasmid pCAX APPswe/ind or exposed to Aβ (1–42) peptides dissolved in culture media for TEER analysis to detect the role of Aβ toxicity. The ZO-1 expression level in the mono-cultured model showed a significant decrease in the number of cells exposed to Aβ compared to the control (Figure 6A). Furthermore, tight junction proteins in the mono-cultured model revealed that ZO-1, claudin-5, and occludin-1 protein expression levels were significantly down-regulated (Figure 6B). In the co-cultured model exposed to Aβ, the TEER analysis also showed a rapid decline in barrier capability (Figure 6C) and the fluorescent immunohistochemistry results demonstrated the same finding that the tight junction protein expression significantly declined compared to the control (Figure 6D). These findings were further confirmed by analyzing the in vivo AD model established with a stereotaxic injection of Aβ1–42 in C57BL/6N mice. The Western blot analysis of the frontal cortex and hippocampal region of Aβ-injected mice showed that the ZO-1 protein expression level was comparably lower than the control (Figure 6E). Aβ co-localized in specific brain regions, which confirmed Aβ’s toxicity, together with the tight junction protein expression levels observed in the Aβ-induced AD model.

## 3. Discussion

The aim of the present study was to establish and analyze an in vitro BBB model and investigate its application in various BBB dysregulated disorders, such as diabetes, TBI, and AD. In this study, we developed a transwell co-culture model that combined brain endothelial cells (bEnd.3) and astrocytes (C8-D1A). Overall, our co-cultured model, compared to the endothelial-only cell model (mono-culture), showed improved BBB structural and functional integrity, as well as higher tight junction protein levels. Additionally, our co-cultured model demonstrated decreased integrity and tight junction protein levels once exposed to the aforementioned disease environment.

### 3.1. Co-Cultured In Vitro Model Mimics the Structural and Functional Integrity of the BBB

TEER is one of the most accurate non-invasive measures of BBB integrity during various cell growth and differentiation stages. The measurement of electrical resistance represents a quantitative analysis method of barrier integrity and function. This value depends on various important factors, such as temperature, cell passage number, the composition of cell media, and the mechano-electronics used for TEER calculation [34,35]. The TEER values of our co-culture were similar to those reported in a previous study using the same cell line and transwell setup [36]. Additionally, the peak value persisted for 4–5 days before declining, which indicates that this period is optimal for mechanistic studies of the BBB and its role in different diseases. Moreover, this model can also provide an opportunity to detect the permeability of neuro-therapeutic drugs and related molecules.

Moreover, molecules that are not lipid-soluble are not able to pass through the BBB without carrier-mediated transport assistance [6]. Therefore, FITC-dextran is a suitable assay to estimate the passive permeability efficiency of an in vitro BBB model [37]. Our results showed that the co-cultured model provided a more compact and intact barrier than the mono-culture model, which was also shown in another study [38]. This may be due to the fact that C8-D1A astrocyte type 1 cells provided enhanced support to bEnd.3 cells in their building barrier property. Studies have shown that astrocytic end-feet processes adhere to endothelial cells to provide additional physiological support to the BBB and help in cell-to-cell communications, provide structural support to the BBB and enhance the expression of tight junction proteins by intercellular communication [39,40].

Furthermore, a compact monolayer of brain endothelial cells is responsible for the barrier properties of the BBB [41]. Additionally, tight junction proteins are involved in the strong cell-to-cell adhesion in this monolayer and control the integrity and barrier function of the BBB [6,42,43]. However, based on our findings, the co-cultured models showed higher tight junction protein levels compared to the endothelial monolayer, which was also shown in another study [38]. Studies have shown that astrocytes helps to maintain BBB integrity, providing structural support and promote the expression of ZO-1 and occludin [44]. ZO-1 strengthens cell-to-cell adhesion, which controls transcellular and paracellular permeability. The decrease in ZO-1 expression compromises BBB integrity and function and causes leakage of the BBB, which allows the unchecked transport of molecules during disease conditions [45,46,47].

### 3.2. Co-Cultured In Vitro Model Is Ideal to Investigate BBB-Related Disorders

The BBB, as mentioned previously, prevents toxic substances in the blood from crossing into the CNS and filters toxic compounds from the brain [48]. Studies have shown that damages to the BBB play a role in the pathogenesis of several disorders, such as obesity, neuroinflammation, and AD [49,50,51]. In order to investigate the role of BBB integrity in obesity due to a high-fat diet, our co-cultured model was exposed to PA-BSA, which showed decreased tight junction protein levels and TEER resistance. In vivo studies have also shown similar findings in which animals fed a high-fat diet showed decreased tight junction proteins levels in the brain [52,53]. Previously, our group has shown that LPS-induced BV-2 cells show increased levels of allograft inflammatory factor 1 (Iba-1), a marker for microglia, and pro-inflammatory cytokines, including tumor necrosis factor alpha (TNFα), and interleukin 1 beta (IL-1β) [54]. Therefore, this explains the increase in GFAP and validates the presence of pro-inflammatory mediators in the MCM. Furthermore, the traumatic brain injury (TBI) inflammatory response in the lesion site is mainly due to the activation of cytokines and studies have shown that tight junction proteins such as claudin-5, occludin, and ZO-1 decrease after the first few days of TBI [55,56]. Lastly, amyloidosis, which is associated with intracellular and extracellular insoluble aggregates that lead to fiber or plaque aggregation, is a hallmark of many neurodegenerative pathologies, including AD, Huntington’s disease, Parkinson’s disease, etc. [57]. Thus, we mimicked the condition of the disease by treating the cells co-cultured in transwell membranes exposed to Aβ (1–42) or by the overexpression of pCAX APP_swe/ind_ plasmids in neurons, both of which lead to Aβ toxicity [58]. Although it is important to note that we measured the total tight junction protein expression in the frontal cortex and hippocampus region, the findings were in line with several in vivo studies, in which the studies showed decreased tight junction protein levels [59,60,61,62]. Moreover, decreased tight junction protein levels have also been shown in postmortem studies of patients with AD [8,9,61]. Therefore, our co-cultured in vitro BBB model validated the structural and functional integrity BBB damage that occurs in the aforementioned disorders.

Although microvascular endothelial cells line the cerebral capillaries in the BBB, astrocytes are essential cells in the CNS that secrete substances such as neurotransmitters, neuromodulators, hormones and peptides, and growth and inflammatory factors, which can enhance or deteriorate the endothelial cells [63]. As such, pathology-associated inducers affect astrocyte activity and influence the structure of the BBB. For example, a study has shown that in mice, a high-fat diet led to obesity, systemic insulin resistance, dysregulated lipid metabolism, and depressive-like behavior. Additionally, it increased the expression of GFAP, shortened the processes of GFAP^+^ cells, and downregulated the expression of astrocytic neuroplasticity-related proteins, GLAST, GLT-1, and connexin-43 in the hippocampus [64]. Moreover, in another study, mice fed a high-fat diet showed increased production of reactive oxygen species (ROS) and pro-inflammatory and endothelial markers, e.g., TNFα, IL-1β, and vascular cell adhesion molecular 1 (VCAM-1). Furthermore, the mice demonstrated a decrease in the levels of claudin-5 and collagen IV, a type of collagen in the basal lamina, compared to the control mice [65]. Lastly, our group has shown, in multiple studies, that intracerebroventricular (i.c.v) injections of Aβ_1–42_ increased GFAP, inflammatory markers, e.g., nuclear factor-κB (NFκB) TNFα, IL-1β, and oxidative stress markers, e.g., nuclear factor erythroid-related factor 2 (Nrf2) and heme oxygenase 1 (HO1), which indicated that AD-associated pathology also increase astrocyte activity and promote inflammation [66,67,68,69]. Therefore, this may explain the decreased integrity of the tight junction dynamics in our transwell co-cultured model.

Lastly, in addition to the aforementioned simple, inexpensive, and functionality of transwell cultures, the advantages of our transwell co-cultured was its wide application in pathology-induced neurodegenerative disease models, as shown in this study. However, it is important to mention that human cells demonstrate differences in morphology and function compared to non-human cells. Additionally, despite a small pore size of 0.4 μm, uncovered pores could provide diffuse penetration; thus, further tests are required to examine its use for novel brain-targeted drug candidates. Nevertheless, our in vitro model could be used prior to investigating human cell BBB models for neurodegenerative diseases.

## 4. Materials and Methods

### 4.1. Cell Culture

The mouse brain endothelial cell line (bEnd.3) was purchased from American Type Culture Collection (No. CRL-2299, ATCC, Manassas, VA, USA). After cell revival, the cells were maintained in Dulbecco’s Modified Eagle’s Medium (DMEM; GIBCO, Thermo Fisher Scientific, Waltham, MA, USA) supplemented with 10% fetal bovine serum (FBS) and 1% penicillin-streptomycin (10,000 U/mL) of the final volume. Astrocyte type I clone (C8-D1A; No. CRL-2541, ATCC) cells were grown in DMEM with an additional 10% FBS and 1% penicillin-streptomycin (10,000 U/mL) of the final volume. The cells were incubated at 37 °C and 5% carbon dioxide. Microglial-conditioned media was produced according to the previously mentioned protocol with some modifications [70]. Briefly, the mouse microglial cell line BV-2 was cultured to above 75% confluency and was treated with lipopolysaccharides from *Escherichia coli* O111:B4 (50 ng/mL; Sigma-Aldrich, L2630, St. Louis, MO, USA) dissolved in cell culture media. After 24 h, the media were aspirated and centrifuged to remove cells and debris. The supernatant was collected for further use.

### 4.2. Cell Seeding in Transwells

To establish a co-cultured model, bEnd.3 cells were seeded on the apical side of the transwell and C8-D1A cells were seeded on the basolateral side of the transwell. The transwell was placed in an inverted orientation to bring the basolateral side of the transwell upward for astrocyte cell seeding. The cells were allowed to adhere to the lower surface of the transwell for almost 48 h (Figure 1A). The whole procedure was performed in an aseptic environment to avoid contamination during the cell culturing procedures. Lastly, AD-like pathology with three cell line co-cultured models was established in a similar way with the addition of HT22 cells to be seeded on the wells of the plate.

For the Western blot and immunofluorescence analyses, the cells were seeded with some modifications compared to the cells used for the permeability and TEER analyses. In this case, a 100 mm dish with permeable transwell support (Costar Corning, Kennebunk, ME, USA) was used. bEnd.3 cells were seeded on the transwell, while C8-D1A cells were seeded in the dish supplemented with 25 mm cell culture-treated coverslips (SPL life Sciences, Republic of Korea) to detect protein expression by immunofluorescence.

### 4.3. Bovine Serum Albumin-Conjugated Palmitic Acid (PA-BSA) Preparation

Bovine serum albumin-conjugated palmitic acid (PA-BSA) was prepared based on a previous study [25] with some modifications. Briefly, 500 mM PA was dissolved in 1 mL of absolute ethanol with constant shaking at 65–70 °C, until completely dissolved. While 10% BSA solution was prepared by dissolving 1.5 g of FFA-Free BSA in serum-free DMEM media at 37 °C, both the solutions were syringe-filtered under aseptic conditions to remove any undissolved particles. In addition, 10 µL of PA was dissolved in 1 mL of BSA at 55 °C, followed by water sonication to form a soluble PA-BSA and stored at −20 °C. The PA-BSA was incubated at 37 °C in a water bath prior to use.

### 4.4. TEER Measurement

TEER was measured periodically to monitor cell confluence and the development of tight junctions. For the measurement of TEER, voltage and current electrode wires were connected via an electrode adaptor (WPI) to an EVOM2 epithelial voltammeter (Millipore, Burlington, MA, USA). The EVOM2 was adjusted to a 10 mA AC current at 12.5 Hz while measuring resistance. Background resistances (R*_BLANK_*) were subtracted from the total calculated resistance R*_TOTAL_* at each time point and normalized for the area, providing TEER values in Ω.·cm^2^ as in the following equation [12]:

R*_TISSUE_* (Ω) = R*_TOTAL_* − R*_BLANK_*

TEER*_REPORTED_* = R*_TISSUE_* (Ω) × M*_AREA_* (cm^2^)

Each group, including an empty transwell for background resistance (R*_BLANK_*), bEnd.3 monoculture, C8-D1A monoculture, or both bEnd.3 and C8-D1A co-cultures under disease conditions, was processed and analyzed in triplicate according to the experimental condition.

### 4.5. Permeability Assays

Fluorescein isothiocyanate (FITC) permeability assay was performed using 1 mg/mL of FITC dextran 70 (FITC:Glucose = 1:250, average molecular weight 70,000; Sigma-Aldrich) dissolved in Hanks’ balanced salt solution (HBSS). A total of 800 µL of the FITC dextran solution was added to the apical side of the transwell, while the basolateral side was supplemented with HBSS buffer. Every 6 h, 100 µL of the sample solution from the basolateral side of the transwell was collected [34]. The amount of FITC that penetrated the transwell membrane in the presence or absence of the cells was measured by fluorescence detection using a GLOMAX multi-detection system (Promega, Madison, WI, USA). Fluorescence was measured at 485 nm absorbance and 510 nm emission.

Solvent persistence and leakage were analyzed by adding 800 µL of the cell culture media on the apical side of the transwell. Prior to the experiment, the mono- and co-cultured models were incubated in the media and during the experiment, the transwell was moved to a new culture dish and incubated in the incubator to examine the solvent persistence and leakage. A lid covered the dish to maintain moisture inside the membrane throughout the experiment. The volume seeped through the membrane and collected in the basolateral part of the transwell, which was calculated and measured every 30 min. The persistence property of the BBB was analyzed by measuring the volume that remained on the apical side of the transwell after 3 h. The media volumes were calculated every half hour using a 1 or 10 μL pipette.

### 4.6. APPswe/ind Plasmid Transfection

The pCAX APPswe/ind plasmid, a gift from Dennis Selkoe and Tracy Young-Pearse (Addgene plasmid # 30145), was transfected in 75% confluent HT-22 cells in a 6-well cell culturing plate. Transfection was conducted using Lipofectamine 3000 transfection reagent (ThermoFisher, Waltham, MA, USA) according to the manufacturer’s protocol.

### 4.7. Hematoxylin and Eosin (H&E) Staining

To detect the cells’ confluency and verify that the cells remained adherent on the basolateral side of the transwell membrane against gravity, the transwells, after cell seeding and culturing, were stained with hematoxylin and eosin (H&E) after fixation with paraformaldehyde solution. The cells were revived by washing with phosphate buffer saline twice. Then, the cells were dehydrated with 70%, 80%, and 90% ethanol. The slides were then treated with hematoxylin for 5 min, and the excess stain was removed by washing with PBS. The cells were co-stained with eosin for 30–45 s. The cells were observed under a compound microscope after the dehydration and washing steps to remove excess stains.

### 4.8. Electron Microscopy

Cells cultured on the transwell membrane were fixed and processed for electron microscopy. A JSM-7610F scanning electron microscope (JEOL USA, Inc., Peabody, MA, USA) was used to obtain the scanning electron micrographs.

### 4.9. Experimental Animals

The experimental procedures were approved by the Research Ethics Committee of the Department of Applied Life Sciences at Gyeongsang National University, the Republic of Korea. All experiments were performed according to the guidelines and regulations of the research ethics committee. Male C57BL/6N mice (8 weeks old, *n* = 8, 4 mice per group) weighing between 24 and 30 g were purchased from Samtako Bio, Osan, South Korea. The animals were handled as per the recommendations of the Institutional Animal Care and Use Committee (IACUC) of the Division of Applied Life Science, Gyeongsang National University, South Korea. The mice were acclimatized for seven days in an animal care house (4–5 per cage) under standard environmental conditions (temperature, 20 ± 2 °C humidity 40% ± 10%; 12 h light/dark cycle) and were provided with normal pellet food and water ad libitum.

### 4.10. Amyloid Beta (1–42) Preparation

Formation of aggregate-free amyloid beta 1–42 (Aβ_1–42_) peptides was performed using hexafluoroisopropane (HFIP), as previously reported with some modifications [71]. Briefly, 2.217 mL of HFIP was added to 10 mg of peptides using a glass Hamilton syringe (Thomas Scientific, New Jersey, United States.) equipped with a Teflon plunger to reach the final concentration of 1 mM and incubated for 30 min until dissolved completely. HFIP-dissolved Aβ solution was aliquoted into non-silanized microcentrifugation tubes for evaporation to allow a dry thin film of peptide to form; then re-dissolved in DMSO; and diluted with phenol-free F-12 cell culture media (Thomas Scientific, Swedesboro, NJ, USA) to reach the final concentration of 100 µM. F-12 cell media-dissolved Aβ peptides were kept at 4 °C until use.

### 4.11. Intracerebroventricular (ICV) Injection of Aβ Peptides

Aβ_1–42_ peptides were injected according to the previously mentioned protocol [72,73]. Briefly, 1 mg/mL of human Aβ_1–42_ peptide stock solution was prepared and incubated at 37 °C for four days. Aggregated Aβ_1–42_ peptides and the respective vehicle were injected at a concentration of 5 µL/mouse or 0.9% NaCl, 5 µL/mouse, respectively, with the help of a Hamilton microsyringe (−0.2 mm anteroposterior (AP), 1 mm mediolateral (ML), and −2.4 mm dorsoventral (DV) into the bregma). As previously suggested, the animals were anesthetized with a combination of 0.05 mL/100 g body weight of Rompun (xylazine) and 0.1 mL/100 g body weight of Zolitil (ketamine) delivered at a rate of 1 ul/5 min, with the injector remaining intact for 5 min.

### 4.12. Protein Extraction and Western Blot Analysis

Protein extraction and Western blot analysis were performed as previously described [74,75]. Briefly, all the mice were anesthetized and decapitated. Their brains were immediately removed and the cortex and hippocampus were separated carefully and frozen at −80 °C. The cortex and hippocampus were homogenized in a pro-prepTM protein extraction solution (iNtRON Biotechnology, Inc., Sungnam, South Korea). The brain samples were then centrifuged at 13,000 r.p.m. at 4 °C for 25 min. The supernatants were collected and stored at −80 °C. Next, the cell lysate protein concentration was quantified using the Bradford assay (Bio-Rad protein assay kit, Bio-Rad Laboratories, Hercules, CA, USA). Equal amounts of protein (20 µg) were electrophoresed under the same experimental conditions, using 8–12% SDS gels and 1x MES SDS running buffer (Novex, Life Technologies, Kiryat Shmona, Israel) with a broad-range prestained protein marker (Xpert prestained protein marker, GenDEPOT, Texas, United States) as a molecular-size control. Membranes were blocked in 5% (*w*/*v*) skim milk to reduce non-specific binding and then incubated with primary antibodies (1:1000–1:10,000 dilutions) overnight at 4 °C. After reactions with a horseradish peroxidase-conjugated secondary antibody, the proteins were detected using an enhanced chemiluminescence (ECL) detection reagent according to the manufacturer’s instructions (Amersham Pharmacia Biotech, Uppsala, Sweden). The X-ray films were scanned, and the optical densities of the bands were analyzed via densitometry, using the computer-based ImageJ software (version 1.50, Wayne Rasband and contributors, National Institutes of Health, Bethesda, MD, USA).

### 4.13. Immunofluorescence Analysis

Immunofluorescence staining was performed according to a previously described protocol with minor modifications [58,76]. Slides with tissue or cells were washed with 0,1 M PBS twice, followed by incubation with proteinase K for 5 min. For blocking, the slides were incubated for 1 h with 2% normal goat serum and 0.1% Triton X-100 in 0.1 M PBS. After washing with PBS, the slides were incubated with primary antibodies overnight at 4°C and with secondary antibodies, including fluorescein isothiocyanate (FITC) or tetramethylrhodamine (TRITC) (anti-mouse and anti-rabbit), at room temperature for 90 min. The slides were stained with 4′,6-diamino-2-phenylindole dihydrochloride (DAPI) for 8 min and covered with fluorescent mounting media (Dako, Santa Clara, CA, USA) and a glass cover slide before microscopy. Immunofluorescence slides were examined using a confocal laser-scanning microscope (Flouview FV 1000, Olympus, Japan) and the relative integrated density values were recorded.

### 4.14. Antibodies

The antibodies used in the present study included anti-ZO-1 (Invitrogen, Thermo Fisher scientific), anti-Occludin-1 (Invitrogen, Thermo Fisher scientific), anti-Claudin-5 (Invitrogen, Thermo Fisher scientific), anti-Amyloid beta and anti-beta-actin (sc-47778) from Santa Cruz Biotechnology (Dallas, TX, USA).

### 4.15. Statistical Analysis

Statistical analyses were conducted by Prism 8.0.2 (GraphPad, San Diego, CA, USA). A Shapiro–Wilk normality test was conducted before any statistical analysis. The data are presented as the mean ± standard error of the mean (SEM). For statistical analysis, two-tailed t-tests and ANOVA with Bonferroni correction were performed. *p*-values less than 0.05 was considered to be statistically significant.

## 5. Conclusions

The present study established an in vitro model and included analyses of physical properties, permeability assays, TEER evaluations, and tight junction protein expression. Furthermore, the present established model has been used to mimic different brain conditions. Our analyses indicate that this model is an excellent in vitro BBB model that can be applied to multiple disease conditions related to BBB integrity. In the future, this model can be used as a convenient tool to study complex physiological conditions, as well as a mode of action of therapeutic agents in diverse neurological disorders, such as Alzheimer’s disease, Parkinson’s disease, ischemia, TBI, and brain metabolic disorders.

## Data Availability

Not applicable.

## Supplementary Materials

The following supporting information can be downloaded at: https://www.mdpi.com/article/10.3390/ijms24065283/s1.
