# Supplementary material for: Establishing Co-Culture Blood–Brain Barrier Models for Different Neurodegeneration Conditions to Understand Its Effect on BBB Integrity
Source: Int J Mol Sci. 2023 Mar 9;24(6):5283. doi: 10.3390/ijms24065283 (PMC10049378; doi:10.3390/ijms24065283)
Supplement: Supplementary file 1 [file ijms-24-05283-s001.zip › ijms-2243095-supplementary.pdf]

Supplementary Materials

Establishing Co-Culture Blood–Brain Barrier Models for Different Neurodegeneration Conditions to Understand Its Effect on BBB Integrity

Jun Sung Park [1,†], Kyonghwan Choe [1,2,†], Amjad Khan [1], Myeung Hoon Jo [1], Hyun Young Park [2,3], Min Hwa Kang [1], Tae Ju Park [4] and Myeong Ok Kim [1,5,*]

1 Division of Life Science and Applied Life Science (BK21 FOUR), College of Natural Sciences, Gyeongsang National University, Jinju 52828, Republic of Korea
2 Department of Psychiatry and Neuropsychology, School for Mental Health and Neuroscience (MHeNs), Maastricht University, 6229 ER Maastricht, The Netherlands
3 Department of Pediatrics, Maastricht University Medical Center (MUMC+), 6202 AZ Maastricht, The Netherlands
4 Haemato-Oncology/Systems Medicine Group, Paul O'Gorman Leukaemia Research Centre, Institute of Cancer Sciences, MVLS, University of Glasgow, Glasgow G12 0ZD, UK
5 Alz-Dementia Korea Co., Jinju 52828, Republic of Korea
* Correspondence: mokim@gnu.ac.kr; Tel.: +82-55-772-1345 (ext. 2655); Fax: +82-55-772-2656
† These authors contributed equally to this study.

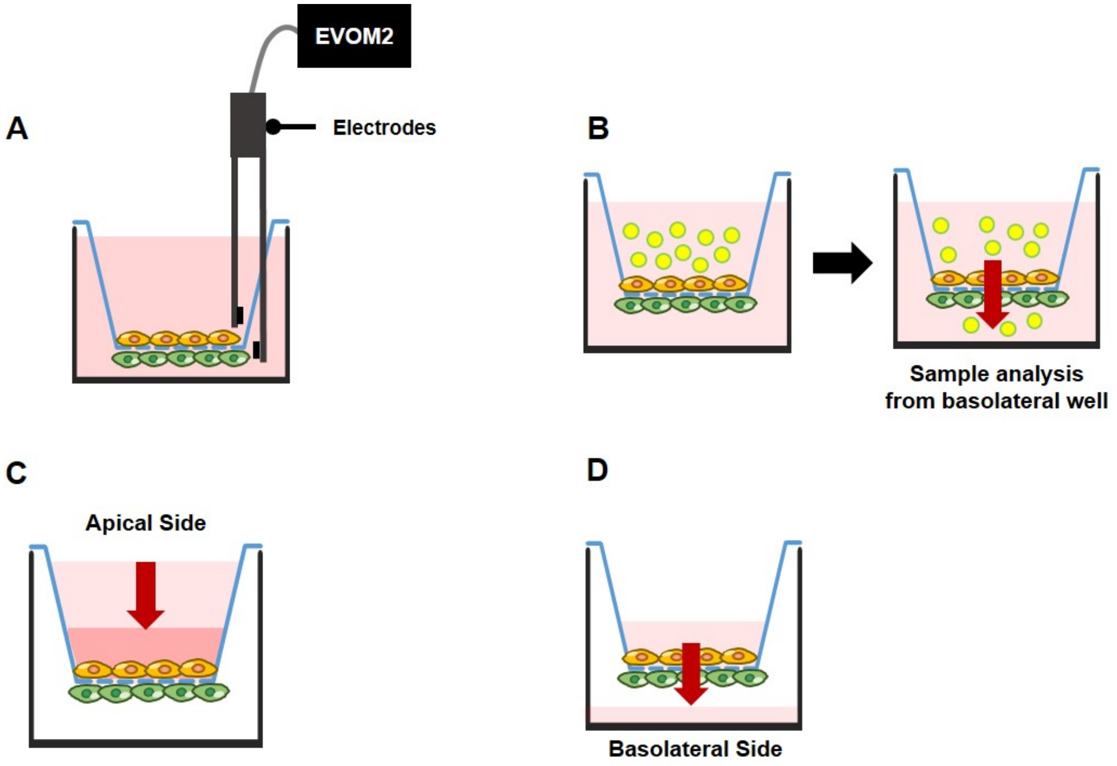

Figure S1. Illustration of the co-culture BBB model setup. (**A**) Transendothelial electrical resistance (TEER) analysis using chopstick electrodes. (**B**) Fluorescein isothiocyanate (FITC) dextran permeability assay. (**C**) Solvent persistence and (**D**) solvent leakage tests.
